# Getting Closer to a Fully Correctable and Connected Research Literature

**DOI:** 10.1371/journal.pmed.1001408

**Published:** 2013-03-26

**Authors:** The PLOS Medicine Editors

## Abstract

The *PLOS Medicine* Editors discuss the need for a dynamic publishing system that enables linkage to corrections of errors in scientific literature (whatever their source) and full integration of articles with post-publication commentary.

Any claim that science is wholly true and definitive (as the media would sometimes have us believe) is nonsense. Current conditions and incentives exist for researchers to engage in poor behavior to advance their careers, establish noteworthy results, gain tenure and eminence, and indeed basically to “publish or perish." At one end of the spectrum is research misconduct, to which there are generally three reactions: deny its existence, chalk it up to a few “bad apples," or accept that it is inevitable and in fact pervasive. This latter approach, which we support, requires that processes are put in place to identify and manage misconduct if prevention is not possible. But there is a bigger challenge: many scientific findings, once thought to be certain, will ultimately be shown to be uncertain by new techniques, a change in thinking, improved data, or the result of a honest error. Unfortunately, changes in the published literature—whatever their origin—simply don't have an adequate paper or electronic trail. Is it time to rethink the correction of the literature?

These topics are at the forefront of recent discussions. On 22 March 2013 the Committee on Publication Ethics (COPE, of which VB is chair), the advisory group for editors, held its European Seminar on the topic of “Publication ethics from student to professional" [1]. The Evidence Live meeting, running between 25 and 28 March [2], includes keynote speeches on research misconduct in the pharmaceutical and medical devices industries and on scientific integrity in scholarly journals.

Taking research misconduct first, we support the approach that recognizes science to be a human activity and thus subjective. Obviously, we acknowledge that misconduct has serious—and objective—implications: for patients who may have been subjected to dubious experimentation, for the reputations of researchers and institutions involved in the misbehavior and the funders who support them, for the whistleblowers who might have risked their own reputations to reveal the misconduct, and for public trust in science and the scientific literature. As a journal, we have developed policies that guide good publication practice and lay out our responsibilities and responses when faced with cases of alleged or proved misconduct, and we follow the guidance of COPE [3].

Furthermore, wherever the misconduct sits on the spectrum—fabrication or falsification of data, plagiarism, breach of ethical standards such as failing to gain adequate informed consent, breaches in publication ethics such as ghost or guest authorship, image manipulation, or fraud, among others—we support approaches that, rather than deny its existence, try to better understand research misconduct: where it exists, what it entails, and how to properly manage it. We publish two articles this week that add to that understanding and reaffirm that, as research is global, so too is the problem of managing research misconduct.

David Resnik and Zubin Master [4] review the state of the art for high-income countries, providing snapshots of the US, Canada, the UK, and Denmark, to illustrate that while many countries have developed policies and initiatives to oversee research integrity, they amount to a patchwork of national, institutional, and professional association level efforts.

Joseph Ana and colleagues [5] offer a complementary view from low- and middle-income countries, analyzing the available data on the prevalence of and response to research misconduct in less-developed countries, where many high-profile cases have emerged and yet little data exist to support the notion that misconduct is more common. Most low- and middle-income countries have yet to develop policies and initiatives to prevent and manage research misconduct, with the exception of China.

What's clear from these two new articles is that research misconduct is a serious issue, that policies and comprehensive responses are patchy, and that more data are needed—not to further establish that misconduct exists, but on how and why it occurs and whether it can be effectively managed and prevented. What's also clear is that while research standards are often considered to be weaker in low- and middle-income countries, any assumption that more misconduct takes place in less-developed settings is misguided and irresponsible. It is a collective responsibility to address this global problem, and all countries and institutions must improve their oversight and management of research misconduct.

The role of journals remains key, and is evolving. While there are helpful resources at our disposal, including those developed by COPE [6], the World Association of Medical Editors [7], and the Council for Science Editors, among others [8], we recognize that misconduct is a timeless issue, the number of publications continues to increase, and the scope and magnitude of post-publication scrutiny grow and are more interconnected. Online commenting, blogging, and social media tools such as Twitter and Facebook, along with global media coverage, have heightened public exposure to research and data and simultaneously to questions about their validity and authenticity.

While the increased scrutiny of research and data should be good for science and medicine, since it inevitably accelerates the pace of scientific and medical discovery (with better outcomes for patients ultimately), what this scrutiny has actually done is expose how disconnected the scientific literature currently is and how far we are from a situation where it can be considered self-correcting.

In fact, the multiple sources of commenting on any given scientific article, it can be argued, lead to more confusion about scientific findings than previously. Scientists are well known to be reluctant to comment specifically on journal articles – the *BMJ* is the only journal that can truly be said to have a vibrant online commenting section. But elsewhere on the web there is proliferation of secondary comments on blogs, Twitter, and other journals. There is now even a Twitter Journal Club [9]. Moreover, there are sites that actively publicize and discuss corrections and retractions, the most well-known of which is Retraction Watch [10]. Yet not many of these secondary articles or posts link back to the original, and in very few cases do articles themselves link out to their post-publication commentary.

It's easy in the face of all this disconnected literature to accept that disconnection is inescapable. However, Crossref [11] and other citation-linking resources already provide some ways of tracing commentary and influence of an article in the context of the evolving scientific field. There are other hopeful developments. The establishment of the “article of record" and any associated corrections are already well in hand with the development of CrossMark [12], which electronically tags articles as that of the “definitive" version and allows the PDF to be linked to corrections from the publisher. It's surely not impossible to develop ways of tagging tweets or other forms of social media that can be linked back and electronically attached to an article, or parts of an article.

Given that the scientific literature is no longer primarily print based, perhaps it is now time to think beyond formal corrections and even retractions of articles (since retracted articles do not disappear from the literature), and to consider how errors in papers (from whatever source), comments, and all the post-publication evolution of papers can be properly linked.

It has never been clearer that the scientific and medical literature is a vibrant, evolving, but imperfect ecosystem. If we can build a system that reflects that dynamism, enables linking to corrections of errors or evolving thinking from whatever source, and allows full integration of articles with post-publication comments of all sorts, then perhaps the new technologies that the web enables can begin to really enhance the literature rather than confuse it, and thereby lead to a fully connected and correctable research literature.
